# Text-prompted foundation-model segmentation of intrapartum fetal ultrasound: a controlled methodological study with F-TPA-SAM2

**DOI:** 10.3389/fmed.2026.1889351

**Published:** 2026-07-28

**Authors:** Zhiyuan Yang, Qinghua Meng, Bentao Jiang, Dongren Liu, Jing Qiu

**Affiliations:** 1 Tianjin University of Sport, Tianjin, China; 2 Jinan Third People's Hospital, Jinan, China; 3 Shandong Sports Rehabilitation Research Center, Jinan, China; 4 Shandong Provincial Maternal and Child Health Care Hospital Affiliated to Qingdao University, Jinan, China

**Keywords:** fetal ultrasound, foundation model, intrapartum monitoring, parameter-efficient adaptation, SAM 2, segmentation, text prompt

## Abstract

**Background:**

Segmenting intrapartum fetal anatomy on B-mode ultrasound supports labor-progress monitoring, but conventional workflows require task-specific models and manual geometric prompts. We investigate whether text-conditioned prompting can select anatomical targets under controlled-label budgets.

**Methods:**

We propose F-TPA-SAM2, a Text-to-Prompt Adapter with 0.871 M trainable parameters (0.135% of the model), which maps frozen FetalCLIP embeddings to a low-rank dense prompt for the frozen SAM 2 mask decoder. We trained the adapter on PSFHS using three seeds and label budgets *N*∈{20, 100, 400}. Controls included no-text and sparse-only variants, blank and wrong-class prompts, a parameter-matched no-text adapter, and fixed random class codes. A supervised U-Net was trained from scratch under the same *N* = 100 and *N* = 400 budgets. Comparisons used image-clustered paired bootstrap confidence intervals and permutation tests. We also assessed zero-shot transfer to 779 HC18 images and compared FetalCLIP, BiomedCLIP, and OpenCLIP at *N* = 100.

**Results:**

On the PSFHS test set, F-TPA-SAM2 achieved 0.811 ± 0.011 Dice at *N* = 100 and 0.859 ± 0.004 at *N* = 400. Differences from the parameter-matched no-text adapter and the fixed random class codes were unclear at both budgets because all paired 95% confidence intervals included zero. F-TPA-SAM2 exceeded U-Net by 0.053 Dice at *N* = 100 (95% CI 0.006–0.085), whereas the *N* = 400 difference was unclear. Wrong-class prompts redirected predictions to the opposite class: requested-class Dice was 0.0003, whereas opposite-class Dice was 0.859 ± 0.003. Zero-shot HC18 Dice was 0.812 ± 0.157, and the three text encoders were within 0.009 Dice.

**Conclusion:**

F-TPA-SAM2 uses a stable category-conditioned input to select between the two trained anatomical targets. However, matched no-text and random-code controls indicate that pre-trained language semantics are not necessary for this result. The findings do not establish open-vocabulary or multi-anatomy segmentation; clinical utility requires a sonographer-in-the-loop and prospective validation.

## 1 Introduction

Accurate segmentation of anatomical structures in obstetric ultrasound (US) is fundamental to routine prenatal and intrapartum care. Fetal head circumference (HC) measurements guide gestational-age estimation (1); pubic-symphysis-to-fetal-head (PS-FH) distance and the angle of progression (AoP) inform labor-monitoring decisions (2, 3); and placental or fetal-cardiac segmentation supports risk stratification. In a real-time intrapartum examination, the sonographer continuously sweeps the probe to locate standard planes, making it impractical to pause and draw a geometric prompt (point or bounding box) on each frame; a language-level command such as “segment fetal head” or “segment pubic symphysis” would be a more natural human–machine interface, set by the protocol or triggered by voice without interrupting the scan.

Conventional supervised models can perform well on a fixed target, but each new anatomical structure typically requires task-specific labels, training, validation, and deployment. This limits the scalability of dataset-specific fetal-US pipelines such as MiTU-Net (4), FM-DACL (5), and Synergistic-FM (6).

These fetal-US methods address different comparisons. MiTU-Net is a task-specific PS–FH segmentation pipeline and is the closest application-level prior, whereas FM-DACL and Synergistic-FM use foundation models in semi-supervised fetal-cardiac settings. Their published results cannot be transferred to our experiment because the anatomical targets, datasets, supervision regimes, and splits differ. We therefore use them to position the application domain, while reserving quantitative head-to-head inference for the matched U-Net trained on our PSFHS split and label budgets.

Vision foundation models offer a potential escape. The Segment Anything Model (SAM) (7) and its video successor SAM 2 (8) provide a powerful prompt-conditioned segmentation backbone, although direct application to medical images underperforms compared to natural images (9). A family of medical adaptations—MedSAM (10), SAM-Med2D (11), Med-SA (12), BALR-SAM (13), and PA-SAM (14)—demonstrates that freezing or lightly tuning the backbone while providing geometric prompts can achieve strong performance across medical modalities, but all of them retain the geometric prompt interface, which is impractical during real-time intrapartum scanning.

A complementary line of work—the text-guided medical SAM family—replaces geometric prompts with text. MedCLIP-SAM (15) and MedCLIP-SAMv2 (16) fine-tune BiomedCLIP and generate SAM prompts via saliency-based pipelines, and are evaluated on breast tumor ultrasound, brain MRI, and lung X-ray. DescriptorMedSAM (17) fuses multi-aspect CLIP text descriptors with MedSAM through cross-attention on the FLARE 2022 abdominal CT benchmark. SEG-SAM (18) uses semantic text enhancement together with point or box prompts and a SAM-style decoder. Grounding DINO-US-SAM (19) combines a LoRA-tuned Grounding DINO with SAM 2 across 18 ultrasound datasets covering breast, thyroid, liver, prostate, kidney, and paraspinal muscle. TextSAM-EUS (20) applies BiomedCLIP-based text prompt learning with a LoRA-adapted SAM for pancreatic tumor segmentation in endoscopic ultrasound. Despite this growing body of study, two gaps remain: (a) no member of this family has been evaluated on *fetal* ultrasound; and (b) none reports a label-budget-controlled cross-dataset evaluation. The closest in spirit is Grounding DINO-US-SAM, but its 18 datasets do not include fetal anatomy.

A critical reading of the text-guided medical SAM family reveals several constraints that motivate our design. MedCLIP-SAM and MedCLIP-SAMv2 rely on a saliency map produced by a text encoder. When that encoder has not been exposed to fetal ultrasound during pre-training, the resulting spatial prompt can miss small or low-contrast anatomy. Grounding DINO-US-SAM introduces a detection stage that requires either bounding-box annotations or a detector pre-trained on the target domain, adding both data and latency overhead. DescriptorMedSAM and SEG-SAM tune or replace SAM's decoder, which increases the number of trainable parameters and complicates deployment on clinical hardware. TextSAM-EUS adapts SAM itself via LoRA, coupling the text-prompt mechanism to a modality-specific tuned backbone. None of these methods has been evaluated on fetal ultrasound, and no reports a label-budget-controlled cross-dataset protocol. We therefore do not claim a fundamentally new segmentation architecture; instead, we position F-TPA-SAM2 as a deliberately minimal implementation with architectural choices subservient to a controlled experimental question.

These methods reduce direct geometric interaction to varying degrees, but they are not uniformly geometry-free: MedCLIP-SAM variants derive a box from saliency, Grounding DINO-US-SAM derives a box from a detector, and SEG-SAM still accepts a point or a box. Their reported results also indicate residual domain sensitivity. Grounding DINO-US-SAM reports a representative DSC of 91.68% on a seen dataset but 64.99%–86.44% on three unseen datasets; TextSAM-EUS reports 82.69%±15.14% DSC with automatic text prompting; and MedCLIP-SAM reports zero-shot DSC of 64.49%–67.82% across its three test datasets. These values are not directly comparable, but they show that text input does not eliminate localization variability. Fetal ultrasound combines small targets, speckle noise, acoustic shadowing, and variability in gestational age and probe position. This motivates evaluating a minimal, frozen-backbone, geometry-free adapter under controlled label budgets rather than assuming that a text interface alone resolves domain shift.

Several methods in this rapidly developing literature were published in 2025–2026, and some remain available only as preprints. Their reported results have not necessarily undergone independent validation and are therefore used here to map the emerging design space rather than as settled comparative evidence. Where a peer-reviewed version was available, we cite that version.

These gaps motivate a focused methodological question. If text is only a convenient label token, then a no-text learned prompt should work just as well, and a wrong prompt should not systematically change the target. If text is a meaningful control signal, then the segmentation target should follow the prompt:

*Under fixed-label budgets, are text prompts actually used to produce intrapartum fetal ultrasound segmentations, or does the adapter silently learn a class-agnostic shape template that ignores the text input?*

To make this question testable, we construct **F-TPA-SAM2** (Fetal Text-to-Prompt Adapter on Frozen SAM 2), a deliberately minimal implementation. A frozen FetalCLIP (21) text encoder maps anatomical names (“fetal head,” “pubic symphysis”) to a 768-dimensional embedding, which is then decoded via a lightweight text-conditioned, low-rank dense-prompt adapter (0.871 M trainable parameters, 0.135% of the combined model) into a frozen SAM 2 mask decoder. The low-rank factorization is not introduced to maximize accuracy; it is a capacity constraint that limits, but does not eliminate, direct generation of class-specific spatial templates from the text vector. The architecture alone cannot establish that pre-trained text semantics are necessary. We address that question empirically using parameter-matched and non-semantic controls. Table 1 summarizes the principal text-guided medical SAM adaptations and their relevance to the present study.

**Table 1:** Comparison of text-guided medical SAM adaptations.

Method	Prompt and modality	Evaluation size	Reported result and relevance
MedCLIP-SAM	Text saliency → box; US/MRI/X-ray	3 datasets; test *n* = 1, 455	Zero-shot DSC 64.49%–67.82%; not fetal US; saliency-dependent
MedCLIP-SAMv2	Text saliency → box; US/MRI/X-ray/CT	4 datasets; test *n* = 3, 470	Mean zero-shot DSC 77.61%; not fetal US; saliency-dependent
DescriptorMedSAM	Text descriptors + cross-attention; no geometry; CT	FLARE22; 50 CT volumes, 12 organs	Full-supervision mean Dice 0.9405; decoder tuning; not fetal US
SEG-SAM	Semantic text + point/box; 8 modalities	Med2D-16M: 3.6M images, 16M masks	Binary DSC 73.15% (point), 81.46% (box); geometric prompt required
Grounding DINO-US-SAM	Text → automatic detector box → SAM 2; US	18 datasets; 12,924 train, 3,881 val, 2,808 unseen test	Representative DSC 91.68% seen; 64.99%–86.44% unseen; detector-dependent
TextSAM-EUS	BiomedCLIP text + LoRA-SAM; no geometry; endoscopic US	18 patients; 11,363 train, 986 val, 4,185 test images	Automatic-text DSC 82.69%±15.14%; not fetal US; backbone tuning
**F-TPA-SAM2** (ours)	**Text** ** → sparse + low-rank dense prompt; no geometry; fetal US**	**PSFHS: 1,358 images; HC18: 779 images**	**PSFHS Dice 0.859; HC18 Dice 0.812; two trained classes only**

Dataset sizes and performance are transcribed from the cited studies and are not directly comparable because modalities, splits, targets, supervision, and prompts differ.

Our contributions are threefold:

1. A **controlled methodological protocol** for evaluating category-conditioned target selection. It combines the original architectural and inference-time prompt controls with a parameter-matched no-text control, fixed random class codes, a matched supervised U-Net, three seeds, and image-clustered paired inference. The controls distinguish the use of a stable category code from necessity of pretrained language semantics.
2. **F-TPA-SAM2**, a minimal candidate implementation whose low-rank dense-prompt design constrains adapter capacity, and whose three-encoder ablation (FetalCLIP, BiomedCLIP, and OpenCLIP ViT-L/14) characterizes the dependence on fetal-specific pre-training. We classify this contribution as *methodological and application-oriented* rather than as a fundamentally new segmentation architecture.
3. A **label-budget-controlled cross-dataset evaluation protocol** transferring fetal-head segmentation from intrapartum PSFHS to prenatal HC18 (22) with no fine-tuning and explicit per-image variance reporting.

We explicitly scope this study to the two foreground classes of PSFHS: fetal head and pubic symphysis. The wrong-class control is a swap between two trained class tokens, not an open-vocabulary test; the HC18 transfer evaluates the fetal head only. We therefore do not claim general multi-anatomy or open-vocabulary text-guided segmentation.

We explicitly do not claim architectural superiority over the broader text-guided medical SAM family. Head-to-head reproduction with these baselines under matched protocols, broader open-vocabulary semantic testing, cine-loop propagation, and prospective clinical validation are deferred to follow-up work (see Discussion).

## 2 Materials and methods

### 2.1 Datasets

We use two publicly released fetal ultrasound datasets, both distributed under the CC BY 4.0 license. All data are fully de-identified and required no additional ethics approval at the participating institutions (see *Ethics statement*).

**PSFHS** (Zenodo record 10969427) (23): 1, 358 intrapartum ultrasound frames of size 256 × 256 pixels, each paired with a three-class pixel-level mask (background, pubic symphysis [PS], fetal head [FH]). The dataset originates from the PSFH challenge for automated measurement of the angle of progression and the PS-FH distance. We used a fixed 70/15/15 train/validation/test split (random seed 42), yielding |train| = 950, |val| = 203, |test| = 205 images, each contributing two foreground-class samples (one per class). The public PSFHS release provides only frame-level identifiers; patient or acquisition identifiers are not available. Our split is therefore image-level, and we cannot rule out patient-level, sequence-level, or temporal leakage between training and testing subsets. Reported Dice values may therefore be optimistic relative to a strictly patient-independent evaluation; the direction and magnitude of any bias cannot be quantified from the released metadata.

**HC18** (22) (Zenodo record 1322001): 779 prenatal fetal-head images of size 800 × 540 pixels with elliptical annotations that we fill into binary masks. HC18 is used *exclusively* for cross-dataset inference; we never train on HC18 and report results on all 779 images as the “test set.”

All headline numbers in this paper use the PSFHS *test* split. The validation split is used only as the early-stopping criterion during training.

### 2.2 F-TPA-SAM2 architecture

**Figure 1:**
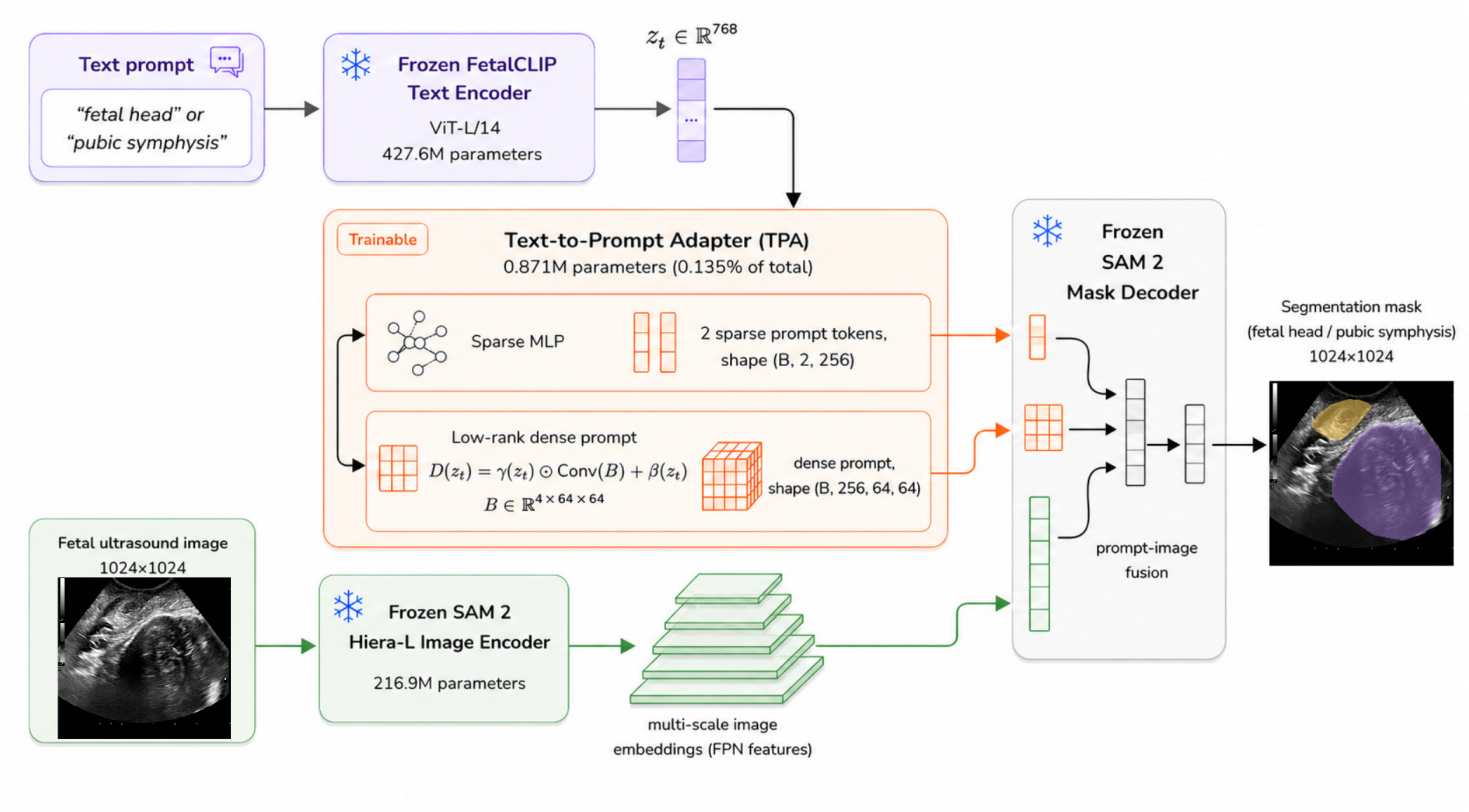
F-TPA-SAM2 architecture. Frozen FetalCLIP text encoder and SAM 2 Hiera-L vision encoder feed the frozen SAM 2 mask decoder via the 0.871 M-parameter Text-to-Prompt Adapter (TPA, red), consisting of a sparse path (two prompt tokens) and a low-rank dense path (Equation 1). Total trainable parameters: 0.135% of 645.4 M. The fetal ultrasound images in Figure were adapted from the PSFHS dataset [Lu et al. (23); Zenodo record 10969427; CC BY 4.0].

F-TPA-SAM2 (Figure 1) consists of three components, only one of which is trainable:

1. A **frozen text encoder** T. We use FetalCLIP (21) (ViT-L/14, context length 117, 427.6 M parameters), which maps a tokenized text prompt **x**_*t*_ to a normalized 768-dimensional vector $z_{t}=T\left(x_{t}\right)\in {\mathbb{R}}^{768}$.
2. A **frozen SAM 2 backbone** (8) consisting of the Hiera-L vision encoder V (216.9 M parameters), the image positional-encoding module, and the mask decoder D. The vision encoder produces a feature pyramid; only the lowest-resolution 256 × 64 × 64 map is used as the image embedding by D.
3. A **trainable Text-to-Prompt Adapter (TPA)** that maps **z**_*t*_ into the (sparse, dense) prompt embeddings expected by D, replacing SAM 2's geometric prompt encoder entirely.

#### 2.2.1 Sparse path

A two-layer MLP with GELU activation maps **z**_*t*_ to two prompt tokens **S**∈ℝ^2 × 256^. These play the role of SAM 2's point-prompt tokens and provide coarse object-level conditioning.

#### 2.2.2 Low-rank dense path

The dense prompt is the critical component. A naive implementation that directly maps **z**_*t*_ through an MLP to the full 256 × 64 × 64 dense prompt would give the adapter enough capacity to memorize a class-specific spatial mask template—exactly the shape-prior failure mode that the present study seeks to exclude. To prevent this, we factor the dense prompt through a fixed-rank learned spatial basis:


$$D\left(z_{t}\right)=\gamma \left(z_{t}\right)⊙{\text{Conv}}_{1\times 1}\left(B\right)+\beta \left(z_{t}\right),\;B\in {\mathbb{R}}^{r\times 64\times 64},$$ (1)

where **B** is a learned spatial basis with rank *r* = 4, Conv_1 × 1_ is a pointwise convolution projecting from *r* to 256 channels (1, 024 parameters), and γ, β:ℝ^768^ → ℝ^256^ are independent two-layer MLPs that predict per-channel scale and shift respectively; ⊙ denotes broadcast element-wise multiplication over the spatial dimensions.

The key constraint is that **z**_*t*_ can modulate the basis only along the *channel* axis and does not directly create new spatial patterns. Any spatial structure in the dense prompt must come from the four learned basis planes, not from the text vector. This design *constrains* direct text-to-spatial-template generation rather than eliminating it entirely; the empirical results below show that the no-text-encoder control (A0) can still reach 0.776 Dice at *N* = 400 by memorizing class templates, but the text encoder provides a consistent additional lift.

Total TPA parameters: 0.871 M, approximately 0.135% of the combined 645.4 M model (FetalCLIP 427.6 M + SAM 2 Hiera-L 216.9 M + TPA 0.871 M). Table 2 reports trainable and total parameters for all methods evaluated in this paper.

**Table 2:** Parameter cost of all methods.

Method	Trainable	Total
F-TPA-SAM2 (FetalCLIP)	0.871 M (0.135%)	645.4 M
A0 FixedLearnedPrompt	2.10 M	219.0 M
A1 ClassPromptTable	0.001 M	216.9 M
SAM 2 box (Oracle)	0	216.9 M
MedSAM box (Oracle)	0	~94 M

“Trainable” is the only set receiving gradients; “Total” includes the frozen text encoder (when applicable) and the SAM backbone. Note the A0 vs. F-TPA-SAM2 trainable mismatch (2.10 M vs. 0.871 M), discussed in Section 4.4.

### 2.3 Architectural controls for falsification

To make the question “does the adapter actually use the text-conditioned input?” formally testable, we train two structural control variants that share F-TPA-SAM2's frozen backbones but remove exactly one design ingredient each:

- **A0–Fixed learned prompt (no text encoder)**. We discard the text encoder entirely and learn a per-class set of sparse tokens (2 × 256) and dense prompt (256 × 64 × 64) as raw parameters, conditioned on the class index rather than text. A0 has 2.10 M trainable parameters. This isolates how much of F-TPA-SAM2's performance can be explained by “two task-specific prompt templates” without any text understanding.
- **A1–Class prompt table (sparse only)**. Same as A0, but the dense prompt is set to zero so that only the two sparse tokens carry class information. A1 has 0.001 M trainable parameters. This isolates whether the dense-prompt path is necessary at all.

The full F-TPA-SAM2 is denoted **A2**. The logical reading is: if A0 achieves the same Dice as A2, the text encoder is unnecessary (the adapter works as a class-template generator); if A1 achieves the same Dice as A0, the dense-prompt path is unnecessary (sparse tokens suffice).

To remove the parameterization confound in A0, we add **A0LR**, a no-text control with the same rank-4 dense-prompt structure as F-TPA-SAM2. A0LR contains 872,704 trainable parameters, compared with 871,168 for F-TPA-SAM2 (0.18% difference), and receives only a learned class index. We also add a **fixed random class-code control**: two distinct, unit-normalized 768-dimensional Gaussian vectors, generated once with control seed 2026, the two classes are assigned and held fixed for all training seeds and during evaluation. This preserves class identity while removing pre-trained language semantics. A fixed two-class label swap is not treated as an independent semantic control because it defines a perfectly learnable reverse code. A0LR and the random class-code control are evaluated at *N*∈{100, 400} with seeds 1–3.

As a matched conventional supervised reference, we train a four-level three-class U-Net (24) from scratch on the same PSFHS split. It uses base width 32, two convolution–BatchNorm–ReLU blocks per level, bilinear decoder upsampling and batch size 4. Its output classes are the background, pubic symphysis, and fetal head. For each budget, it uses *N* annotated training images, equivalent to *N* masks per foreground class, with the selected image identifiers saved for reproducibility. The U-Net uses no external pre-training and is evaluated at *N*∈{100, 400} with seeds 1–3.

### 2.4 Prompt-semantic controls

For *N* = 400 we additionally evaluate F-TPA-SAM2 across the same three training seeds under three prompt-semantic controls (in addition to the normal prompt):

- **Blank**: the text input is an empty string, so **z**_*t*_ is the text encoder's representation of “nothing.”
- **Random**: **z**_*t*_ is replaced by a zero-mean unit-variance Gaussian vector with the same norm as the real embedding.
- **Wrong-class**: the class labels are swapped (“fetal head” ↔ “pubic symphysis”), so the model receives a semantically valid but factually incorrect prompt.

For each control, we report Dice against the requested-class ground truth, predicted mask area (as a fraction of image area), and the empty-mask rate. For the wrong-class control, we additionally report Dice against the *opposite*-class ground truth—the class whose name was fed in after the swap—to test whether the model follows the prompt word to its trained other target.

### 2.5 Geometric-prompt baselines

**SAM 2 box (Oracle)** and **MedSAM box (Oracle)**: we provide the minimum enclosing bounding box of the ground-truth mask as the prompt at test time. This represents the strongest possible geometric prompt and serves as a *potentially optimistic Oracle-prompt reference*: it is not a practical workflow, since the operator would need to know the ground-truth mask to draw the box. It does not constitute a formal performance ceiling. We use the public weights facebook/sam2-hiera-large and wanglab/medsam-vit-base.

### 2.6 External text-guided reference

For an external reference against the closest text-guided medical SAM study, we evaluate MedCLIP-SAMv2 (16) on PSFHS. Because the BiomedCLIP weights released with that work are not finetuned on fetal US, we do not attempt a strict saliency-driven evaluation; instead, we replaced its saliency-driven box with the Oracle GT box and feed it to SAM v1 (vit_h). The resulting Dice should be read as a potentially optimistic, non-comparable reference rather than a fair head-to-head comparison: the two methods use different SAM backbones and different training protocols.

### 2.7 Three-encoder ablation

To probe whether F-TPA-SAM2 depends on fetal-specific pre-training in the text encoder, we train it with three frozen encoders spanning three pre-training domains, all at *N* = 100 with 3 random seeds and otherwise identical hyperparameters: **FetalCLIP** (21) (210, 000 fetal-US image–text pairs, fetal-specific, embedding dimension 768); **BiomedCLIP** (25) (PMC-15M, general medical, embedding dimension 512); and **OpenCLIP ViT-L/14** (26) (LAION-2B, web-general with no medical exposure, embedding dimension 768).

### 2.8 Label budget and seeds

For F-TPA-SAM2 (with FetalCLIP) and the architectural controls A0/A1, we train under three label budgets *N*∈{20, 100, 400} masks per foreground class, randomly sampled from the training split. Each (*N*, method) configuration is run with three random seeds {1, 2, 3}, and we report mean ± standard deviation over seeds. A0LR, the fixed random class-code control and supervised U-Net are evaluated only at the two informative budgets *N*∈{100, 400}; *N* = 20 is omitted because all original variants remain at the no-information plateau at that budget.

### 2.9 Training details

Only the TPA module is trained; both the text encoder and the SAM 2 backbone remain frozen throughout. We used AdamW with a learning rate 10^−4^, weight decay 10^−4^, batch size 4, input size 1, 024 × 1, 024, and bf16 mixed precision. The loss is the sum of the Dice loss and Focal loss (weights 1:1, α = 0.25, γ = 2.0). Data augmentation consists of a random horizontal flip (probability 0.5) and brightness jitter (±10%). Training runs for up to 50 epochs with early stopping triggered after 8 epochs without improvement in validation Dice. All experiments run on a single NVIDIA RTX PRO 6000 Blackwell Server GPU (96 GB).

The supervised U-Net uses the same image size, augmentation, AdamW learning rate, weight decay, maximum epoch count, and early-stopping patience. Its objective is cross-entropy plus foreground multiclass Dice loss; model selection uses validation macro-Dice only.

### 2.10 Evaluation metrics

We report the Dice coefficient (primary), the Hausdorff distance at the 95th percentile (HD95) and average symmetric surface distance (ASSD) as segmentation-quality metrics. For the negative controls, we additionally report predicted mask area ratio and empty-mask rate. For cross-dataset transfer we report per-image Dice variance to surface domain-shift effects rather than hiding them behind a mean.

### 2.11 Statistical analysis

All paired comparisons are matched explicitly by (image_id, class_idx, seed). We estimate the mean paired Dice difference using a two-level paired bootstrap with 10,000 replicates: training seeds are resampled first, followed by resampling the 205 original test images within each selected seed. The two classes Of observations from one image are always resampled together. We report the paired effect size and percentile 95% confidence interval. Primary comparisons are F-TPA-SAM2 versus A0LR, fixed random class codes, and U-Net at *N*∈{100, 400}. Encoder comparisons at *N* = 100 are exploratory. For hypothesis tests, the null is a zero-mean paired Dice difference. We use 100,000 image-clustered paired sign permutations and Holm correction across the six primary comparisons; the two exploratory encoder comparisons form a separate correction family. No inference is based on a single seed or on best-validation values.

## 3 Results

### 3.1 Main result and architectural controls

**Figure 2:**
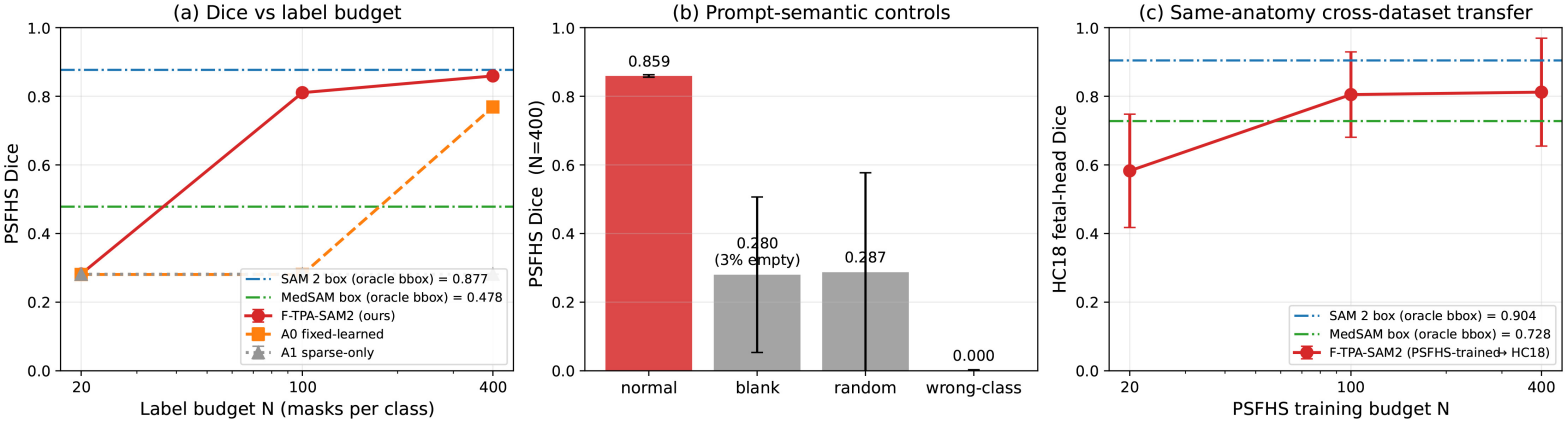
**(a)** PSFHS test Dice vs label budget *N*; horizontal lines are oracle-bbox references (impractical at test time). **(b)** Prompt-semantic controls at *N* = 400. **(c)** Cross-dataset transfer PSFHS → HC18; error bars reflect per-image domain-shift variance.

Table 3 and Figure 2a report the PSFHS test-set Dice across the three label budgets for F-TPA-SAM2 (A2) and the two architectural controls (A0, A1).

**Table 3:** PSFHS test Dice (410 samples; PS, pubic symphysis; FH, fetal head); mean ± std over 3 seeds where applicable.

Method	*N* = 20	*N* = 100	*N* = 400
F-TPA-SAM2 (FetalCLIP, ours)	0.282 ± 0.002	**0.811**±0.011	**0.859**±0.004
A0 (no text encoder)	0.280 ± 0.000	0.280 ± 0.000	0.776 ± 0.004
A1 (sparse only)	0.281 ± 0.000	0.281 ± 0.000	0.281 ± 0.000
Text-guided medical SAM (external Oracle-prompt reference)			
MedCLIP-SAMv2^†^	0.834 ± 0.109 (PS 0.774/FH 0.895)		
Geometric-prompt reference (Oracle GT bbox)			
SAM 2 box (Oracle)	0.877 ± 0.059 (PS 0.858/FH 0.896)		
MedSAM box (Oracle)	0.478 ± 0.233 (PS 0.398/FH 0.558)		

Geometric baselines and MedCLIP-SAMv2^†^ use Oracle GT bounding boxes (impractical at test time) and are reported as *potentially optimistic Oracle-prompt references*, not fair comparisons; F-TPA-SAM2 uses only text at test time. See Section 3.

^†^BiomedCLIP + SAM v1 (vit_h); Oracle GT bbox replaces saliency-driven box (potentially optimistic reference).

Bold values indicate the best Dice among the three trained architectural variants at the informative budgets *N* = 100 and *N* = 400.

F-TPA-SAM2 reaches 0.859 ± 0.004 Dice at *N* = 400 (mean ± std over three seeds), 0.811 ± 0.011 at *N* = 100, and plateaus near 0.282 at *N* = 20 where only 40 training images are available. At *N* = 20 all three methods (A0, A1, A2) produce similar near-random output, consistent with insufficient gradient signal for *any* prompted SAM 2 configuration.

**A1 (two-token sparse-only prompt)** stays at ≈0.281 across *all* label budgets, including *N* = 400. Under our specific design choice—two learnable sparse prompt tokens fed into the frozen SAM 2 decoder without any dense path—sparse-only conditioning is insufficient to localize either fetal structure. We do not claim that all possible sparse-prompt designs would fail; richer sparse-token variants or mixed sparse–dense designs are a natural follow-up.

**A0 (no text encoder)** follows the same plateau at *N* = 20 and *N* = 100 but eventually reaches 0.776 at *N* = 400, where it has enough gradient signal to memorize a per-class prompt template. The gap between F-TPA-SAM2 and A0 is +0.083 Dice at *N* = 400 and much larger at *N* = 100 (0.811 vs. 0.281). This gap should be interpreted with care: A0 has 2.10 M trainable parameters versus 0.871 M for F-TPA-SAM2, and the two modules differ in both parameterization and optimization difficulty. This original A0 comparison does not isolate text encoding from parameterization or capacity, and is therefore not used to attribute a performance benefit to language. The matched controls below supersede that interpretation.

Table 4 reports the reviewer-requested matched controls. A0LR contains 872,704 trainable parameters, only 0.18% more than F-TPA-SAM2 (871,168), and uses the same rank-4 dense-prompt structure without a text encoder. Its Dice is 0.810 ± 0.001 at *N* = 100 and 0.862 ± 0.002 at *N* = 400; neither paired difference from F-TPA-SAM2 is clear because both 95% CIs include zero. Likewise, a control trained with two fixed, distinct, normalized, and non-trainable random vectors reaches 0.814 ± 0.003 and 0.858 ± 0.006; again, both CIs include zero. Thus, in this two-class task, the experiments do not show that pre-trained text embeddings improve segmentation over a parameter-matched non-text mechanism or arbitrary stable category codes. The wrong-class result remains evidence of category-conditioned target selection, but not of pre-trained linguistic semantics.

**Table 4:** Matched reviewer-requested controls on the PSFHS test split.

Method	*N* = 100	*N* = 400
A. Test dice		
F-TPA-SAM2	0.811 ± 0.011	0.859 ± 0.004
A0LR (parameter-matched, no text)	0.810 ± 0.001	0.862 ± 0.002
Fixed random class codes	0.814 ± 0.003	0.858 ± 0.006
Supervised U-Net (from scratch)	0.758 ± 0.032	0.868 ± 0.006
B. Paired difference Δ (95% CI); Holm-adjusted *p*		
F-TPA-SAM2 − A0LR	+0.001 [−0.012, 0.010]; .784	−0.003 [−0.006, 0.001]; 0.125
F-TPA-SAM2 − random codes	−0.004 [−0.014, 0.006]; 0.240	+0.002 [−0.005, 0.011]; 0.403
F-TPA-SAM2 − U-Net	+0.053 [0.006, 0.085]; < 0.001	−0.009 [−0.017, 0.001]; 0.173

Panel A reports mean ± sample SD over three training seeds. Panel B reports the paired Dice difference (F-TPA-SAM2 minus comparator), a two-level 95% bootstrap CI (10,000 replicates; seeds resampled first and 205 original images resampled within seed, with both classes kept together), and Holm-adjusted *p* from 100,000 image-clustered sign permutations across the six primary comparisons.

The supervised U-Net reaches 0.758 ± 0.032 at *N* = 100 and 0.868 ± 0.006 at *N* = 400. F-TPA-SAM2 is higher at the smaller budget by 0.053 Dice (95% CI 0.006–0.085, Holm-adjusted *p* < 0.001). At *N* = 400, U-Net is numerically higher by 0.009, but the paired CI (−0.017 to 0.001 for F-TPA-SAM2 minus U-Net) includes zero and the Holm-adjusted *p* = 0.173. This supports a low-label advantage under the present split, not general superiority to supervised segmentation.

The geometric Oracle-bbox baselines bracket the achievable range: SAM 2 box reaches 0.877 and MedSAM box only 0.478 on PSFHS test (domain shift).

F-TPA-SAM2 trails SAM 2 box (Oracle) by only 0.018 Dice while requiring no spatial input at test time.

For an external reference against the closest text-guided medical SAM family, we also evaluate MedCLIP-SAMv2 (16) on PSFHS. Its public BiomedCLIP weights are not finetuned on fetal US, so we do not attempt a strict saliency-driven evaluation; instead, we replace its saliency-driven box with the Oracle GT box and feed it to SAM v1, which yields 0.834 Dice. We emphasize that this is a potentially optimistic, non-comparable reference: an Oracle-bbox prompt is not the test-time input for either MedCLIP-SAMv2 or F-TPA-SAM2. The two methods use different SAM backbones (v1 vs. v2), and the training protocols differ. The number is contextual only and is not evidence for or against the superiority of either method; a head-to-head comparison would require matched backbones, prompts, splits, and supervision.

Surface-distance metrics tell the same story (Table 5). At *N* = 400 F-TPA-SAM2 achieves HD95 = 52 ± 1 pixels and ASSD = 17 ± 0.4 pixels at 1, 024 × 1, 024 resolution, the lowest of the three trained variants. A1 stays at the no-information baseline across all budgets, and A0 only escapes this baseline at *N* = 400, consistent with the Dice numbers.

**Table 5:** Surface distance metrics on PSFHS test, inter-seed mean±std over 3 seeds (pixel units at 1, 024 × 1, 024).

	*N* = 20		*N* = 100		*N* = 400	
Method	HD95	ASSD	HD95	ASSD	HD95	ASSD
F-TPA-SAM2	558 ± 2	211 ± 2	74 ± 3	23 ± 1	**52 ± 1**	**17 ± 0.4**
A0 (no text enc.)	560 ± 0	214 ± 0	560 ± 0	214 ± 0	89 ± 1	28 ± 0.4
A1 (sparse only)	560 ± 1	214 ± 1	560 ± 1	215 ± 1	561 ± 1	215 ± 0.5

HD95, 95th-percentile Hausdorff distance; ASSD, average symmetric surface distance. Large values at *N* = 20 reflect the no-information baseline (near-empty masks). At *N* = 400, F-TPA-SAM2 has the lowest surface error. Bold values indicate the lowest (best) surface-distance values at *N* = 400.

Per-seed test and validation Dice for all methods are reported for transparency in Tables 6, 7 (Sec. 3.6).

**Table 6:** Per-seed PSFHS *test-split* Dice for all methods and label budgets.

Method, *N*	Seed 1	Seed 2	Seed 3
F-TPA-SAM2, *N* = 20	0.2806	0.2814	0.2841
F-TPA-SAM2, *N* = 100	0.8149	0.7982	0.8187
F-TPA-SAM2, *N* = 400	0.8628	0.8552	0.8603
A0LR, *N* = 100	0.8094	0.8105	0.8101
A0LR, *N* = 400	0.8638	0.8611	0.8611
Fixed random codes, *N* = 100	0.8172	0.8123	0.8131
Fixed random codes, *N* = 400	0.8517	0.8579	0.8638
Supervised U-Net, *N* = 100	0.7460	0.7942	0.7339
Supervised U-Net, *N* = 400	0.8626	0.8666	0.8747
A0 FixedLearnedPrompt, *N* = 20	0.2801	0.2802	0.2801
A0 FixedLearnedPrompt, *N* = 100	0.2800	0.2802	0.2802
A0 FixedLearnedPrompt, *N* = 400	0.7737	0.7810	0.7730
A1 ClassPromptTable, *N* = 20	0.2802	0.2807	0.2811
A1 ClassPromptTable, *N* = 100	0.2806	0.2810	0.2808
A1 ClassPromptTable, *N* = 400	0.2804	0.2814	0.2808

Means and standard deviations of these numbers are the values reported in Table 3.

**Table 7:** Per-seed PSFHS *validation* Dice (early-stopping criterion only).

Method, *N*	Seed 1	Seed 2	Seed 3
F-TPA-SAM2, *N* = 20	0.2812	0.2818	0.2847
F-TPA-SAM2, *N* = 100	0.8084	0.7964	0.8105
F-TPA-SAM2, *N* = 400	0.8528	0.8472	0.8548
A0 FixedLearnedPrompt, *N* = 20	0.2807	0.2808	0.2807
A0 FixedLearnedPrompt, *N* = 100	0.2804	0.2808	0.2807
A0 FixedLearnedPrompt, *N* = 400	0.7674	0.7748	0.7641
A1 ClassPromptTable, *N* = 20	0.2808	0.2814	0.2819
A1 ClassPromptTable, *N* = 100	0.2813	0.2817	0.2815
A1 ClassPromptTable, *N* = 400	0.2811	0.2818	0.2815

Headline test numbers are in Table 3; per-seed test numbers in Table 6.

**Figure 3:**
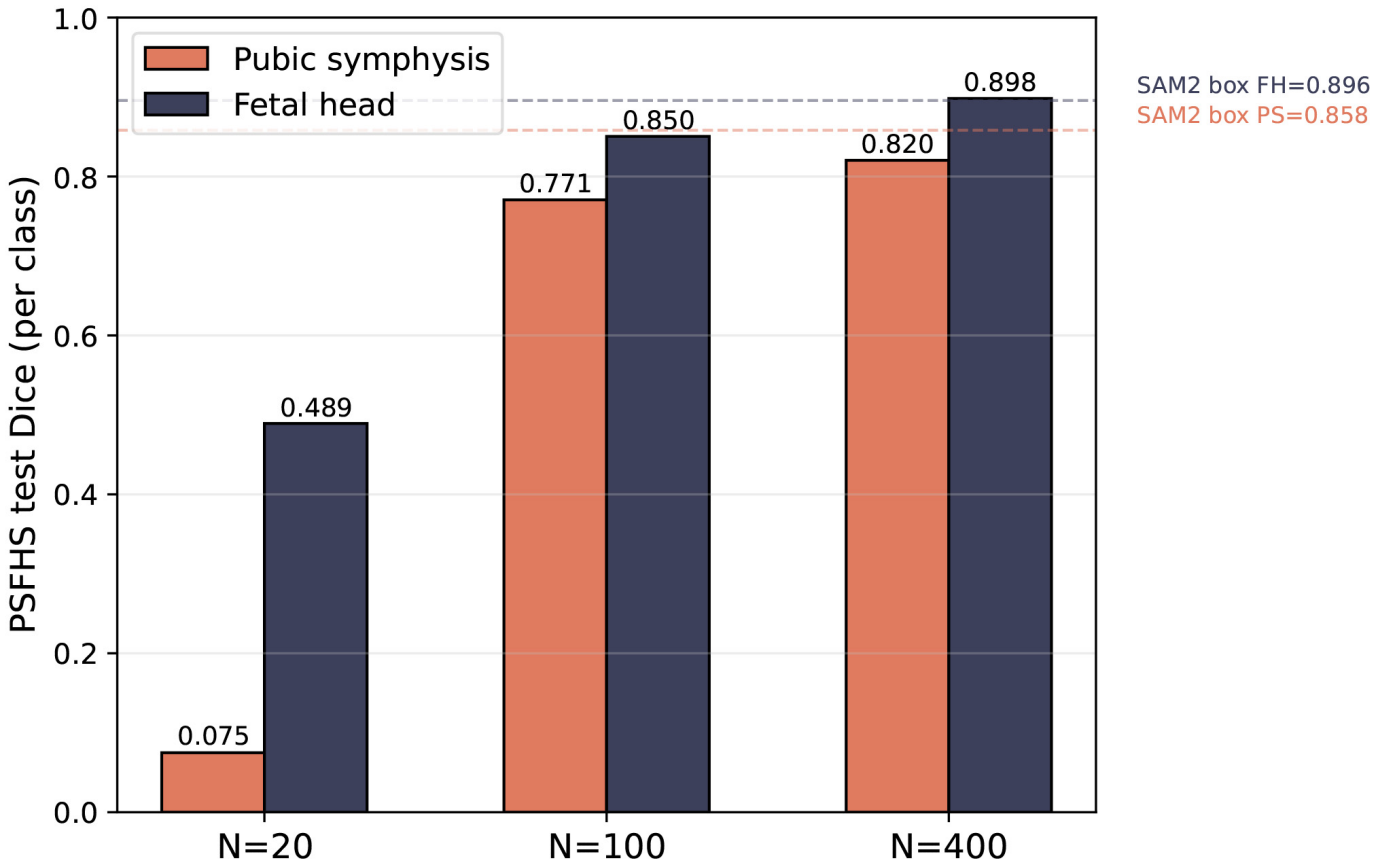
Per-class PSFHS test Dice for F-TPA-SAM2 (FetalCLIP). Pubic symphysis (PS) is consistently harder than fetal head (FH) across all label budgets, consistent with the Oracle-bbox SAM 2 reference (dashed lines).

Figure 3 breaks down the PSFHS test Dice by foreground class. PS is consistently harder than FH across all *N* (0.820 vs. 0.898 at *N* = 400), mirroring the per-class gap seen in the Oracle-bbox SAM 2 baseline (0.858 vs. 0.896). The smaller, more deformable PS is a challenging target for both text- and bbox-prompted methods.

### 3.2 Is the text actually being used?

Table 8 and Figure 2b present the prompt-semantic negative controls at *N* = 400.

**Table 8:** Prompt-semantic negative controls on PSFHS test with F-TPA-SAM2 *N* = 400, inter-seed mean ± std over 3 seeds.

Prompt mode	Dice (vs. req. GT)	Empty rate	Pred area	Dice (vs. opp. GT)
Normal	0.859 ± 0.004	—	11.3%	—
Blank ("")	0.267 ± 0.009	7.2%	54.2%	—
Unseen random embedding (inference only)	0.305 ± 0.018	0.0%	18.9%	—
Wrong-class	0.0003 ± 0.0001	0.0%	11.4%	**0.859 ± 0.003**

The last column is Dice against the *opposite*-class GT—i.e., the class whose name was fed in after the swap. Wrong-class prompts match opposite-class GT at 0.859 Dice, identical to the normal-prompt Dice on the requested target. See Section 3.2.

req., requested-class GT; opp., opposite-class GT.

At *N* = 400, replacing the correct prompt with a wrong-class prompt collapses Dice from 0.859 to **0.0003**. The predicted mask area (11.5%) closely matches the ground-truth area (11.3%), and the empty-mask rate is 0%, so the model is not “giving up.” To test whether the prediction in fact follows the swapped prompt word to its trained other target, we also compare each wrong-class prediction against the *opposite*-class ground-truth mask (the class whose name was actually fed in). The result is striking (Table 8, last column): mean Dice against the opposite-class GT is **0.859 ± 0.003**—identical to the normal-prompt Dice on the requested target (0.859), and broken down per class, 0.898 ± 0.001 (prompt = “fetal head,” opposite GT = FH) and 0.820 ± 0.007 (prompt = “pubic symphysis,” opposite GT = PS), which again match the normal-prompt per-class Dice of 0.898/0.820. Across all seeds the opposite-Dice exceeds the requested-Dice on **100%** of images, and exceeds 0.7 on 94.96%. Under our two trained classes, this is evidence that the adapter uses its category-conditioned input to select between trained anatomical targets rather than learning a single class-agnostic shape prior. It is not evidence that pre-trained language semantics are necessary: Table 4 shows that fixed random class codes support comparable segmentation.

**Figure 4:**
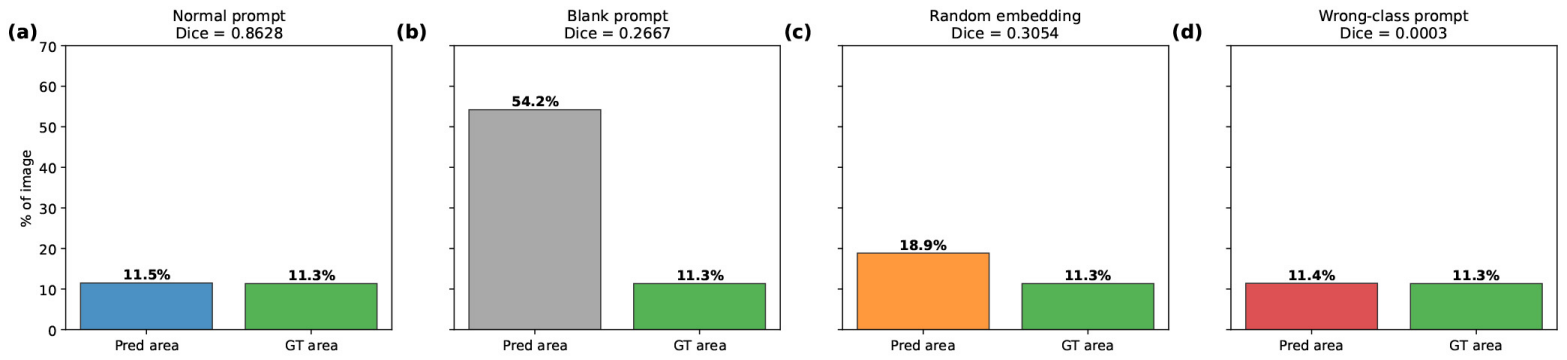
Predicted mask area versus ground-truth area under each prompt control (*N* = 400): **(a)** normal prompt; **(b)** blank prompt; **(c)** random embedding; and **(d)** wrong-class prompt. Wrong-class prompts produce masks of the correct size on the wrong structure (Dice ≈ 0); blank prompts trigger over-segmentation (~54% of the image); and random embeddings yield unfocused masks (~19%).

Blank prompts trigger over-segmentation: averaged over the three seeds, the predicted mask covers 54.2% of the image (vs. 11.3% ground truth), yielding Dice 0.267 ± 0.009—the model defaults to a “segment everything” mode when given no discriminative text signal. Random Gaussian embeddings introduced only at inference, and therefore unseen during training, produce smaller but unfocused masks (18.9% area, Dice 0.305 ± 0.018). This prompt-corruption experiment is distinct from the trained fixed-random class-code control in Table 4. Figure 4 visualizes predicted vs. ground-truth mask area for each control, making the mechanism behind each failure mode immediately apparent.

### 3.3 Encoder ablation across three pretraining regimes

To quantify whether the text encoder is a critical bottleneck for F-TPA-SAM2, we train three frozen text encoders that span three qualitatively different pretraining domains: **FetalCLIP** (210k fetal-US image–text pairs, fetal-specific), **BiomedCLIP** (PMC-15M, general medical), and **OpenCLIP ViT-L/14** (LAION-2B, web-general, and no medical exposure). All other components (TPA architecture, learning rate, schedule, and label budget) are held identical at *N* = 100 with 3 seeds each. The PSFHS test Dice values are 0.811 ± 0.011 (FetalCLIP), 0.802 ± 0.015 (BiomedCLIP), and 0.809 ± 0.008 (OpenCLIP).

All three means fall within a 0.009-Dice window. In the pre-specified exploratory paired analysis, FetalCLIP exceeds BiomedCLIP by 0.009 Dice (95% CI 0.003–0.015; Holm-adjusted *p* < 0.001), whereas its 0.002 difference from OpenCLIP is unclear (95% CI −0.003–0.007; Holm-adjusted *p* = 0.245). The FetalCLIP-BiomedCLIP difference is statistically distinguishable under this split, but its magnitude is small. By contrast, the FetalCLIP-OpenCLIP difference is unclear; together with the fixed-random-code results, this indicates that fetal-specific language pretraining is not required for competitive two-class segmentation. We therefore describe the model as category-conditioned rather than as demonstrating deep medical language semantics.

**Figure 5:**
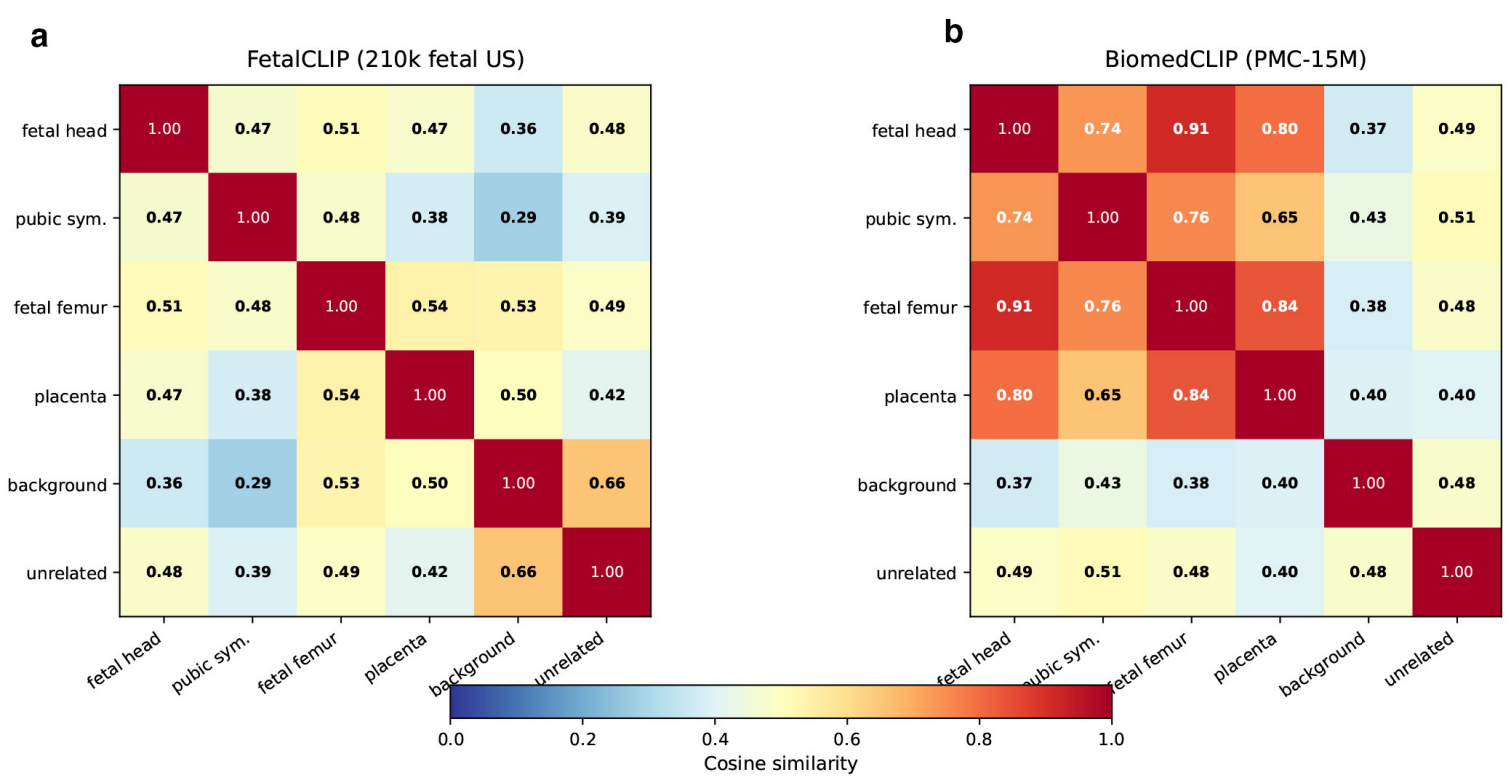
Cosine similarity matrices of text embeddings for six anatomy-related prompts. **(a)** FetalCLIP (fetal-specific) gives a cosine similarity of 0.47 between “fetal head” and “pubic symphysis”; **(b)** BiomedCLIP gives a similarity of 0.74. OpenCLIP was evaluated in the downstream segmentation ablation (Section 3.3) but is not visualised here. At *N* = 100, FetalCLIP exceeded BiomedCLIP by 0.009 Dice.

Figure 5 provides a complementary view of the embedding spaces for FetalCLIP and BiomedCLIP. FetalCLIP's “fetal head” embedding has a cosine similarity of 0.47 to “pubic symphysis”, whereas the corresponding BiomedCLIP similarity is 0.74. OpenCLIP was included in the downstream segmentation ablation but is not visualised in Figure 5. Despite the sharper separation in FetalCLIP's embedding space, FetalCLIP exceeded BiomedCLIP by only 0.009 Dice. This embedding-space observation is descriptive and does not establish a causal relation between cosine separation and segmentation. Characterising additional encoders and larger class vocabularies is left to future work.

### 3.4 Same-anatomy cross-dataset transfer

Table 9 and Figure 2c report Dice on HC18 fetal-head segmentation when F-TPA-SAM2 is trained only on PSFHS and applied to HC18 with no fine-tuning.

**Table 9:** Same-anatomy cross-dataset transfer: F-TPA-SAM2 trained on PSFHS (intrapartum) tested on HC18 (prenatal fetal head, *N*_test_ = 779).

>PSFHS training budget	HC18 Dice	*N*_test_
*N* = 20	0.583 ± 0.165	779
*N* = 100	0.805 ± 0.124	779
*N* = 400	0.812 ± 0.157	779
SAM 2 box (Oracle, no training)	0.904 ± 0.188	779
MedSAM box (Oracle, no training)	0.728 ± 0.181	779

The high std reflects a genuine domain shift, not a single mean Dice; see Figure 7 for the per-image distribution.

**Figure 6:**
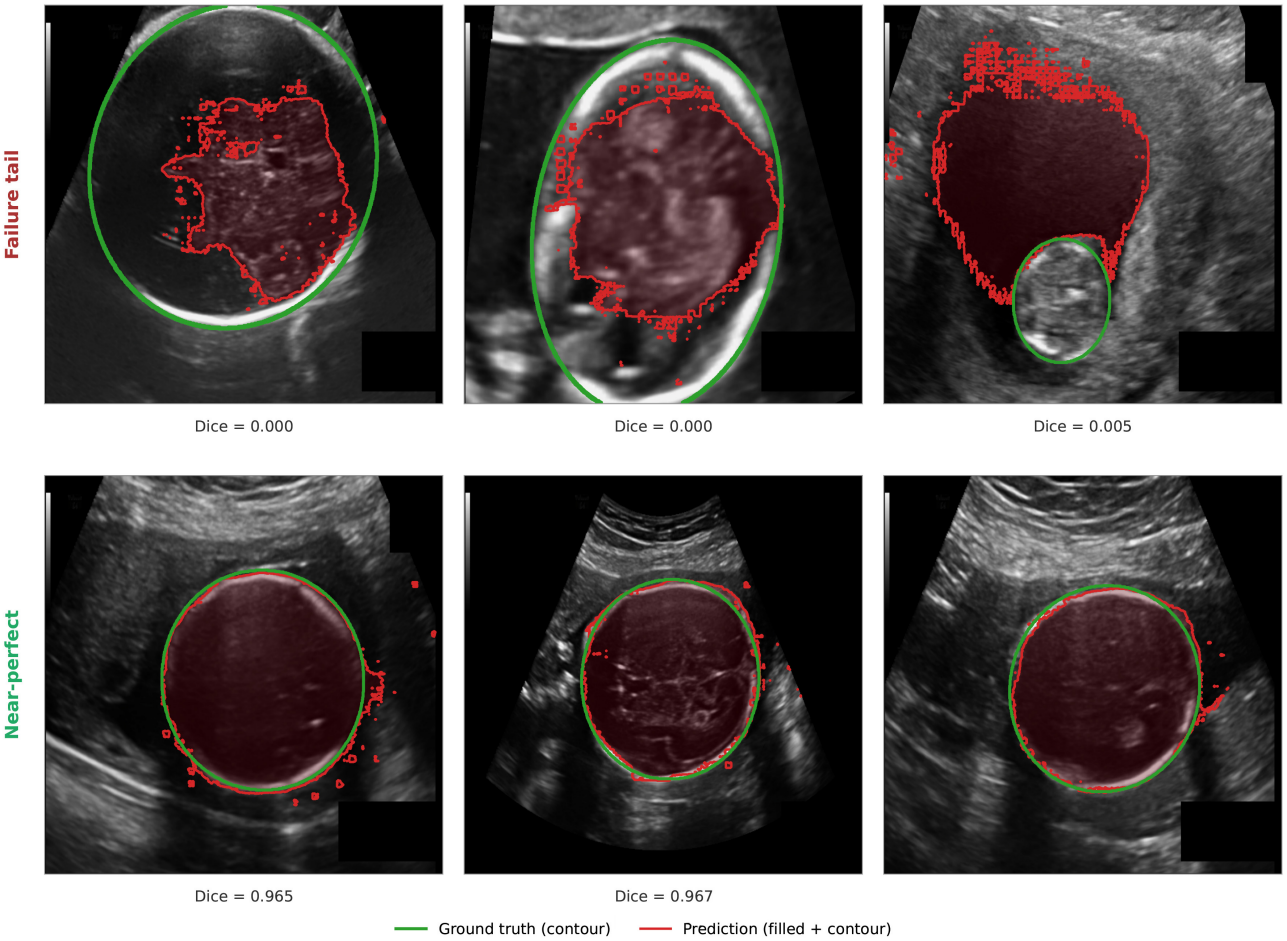
HC18 cross-dataset failure analysis. **Top**: three worst Dice cases (atypical orientation or low contrast). **Bottom**: three best-Dice cases. Green contour, ground truth; red mask + contour, F-TPA-SAM2 prediction. The Bimodal behavior motivates per-patient quality control. The ultrasound images in Figure were adapted from the HC18 dataset [van den Heuvel et al. (22); Zenodo record 1322001; CC BY 4.0].

At *N* = 400, the mean Dice is 0.812 ± 0.157. The mean Dice of 0.812 is encouraging, as it exceeds the performance of the MedSAM box with Oracle bounding boxes (0.728). However, the large standard deviation is itself a finding: prenatal HC18 imaging conditions differ substantially from intrapartum PSFHS, and on a non-trivial subset of HC18 images even F-TPA-SAM2 produces a poor mask. Figure 6 illustrates this bimodal behavior: the three worst HC18 cases achieve Dice ≤ 0.001 (complete segmentation failure, likely due to atypical head orientation or low contrast), while the three best cases achieve Dice >0.96 (near-perfect). This failure tail warrants per-patient quality control in any clinical deployment.

**Figure 7:**
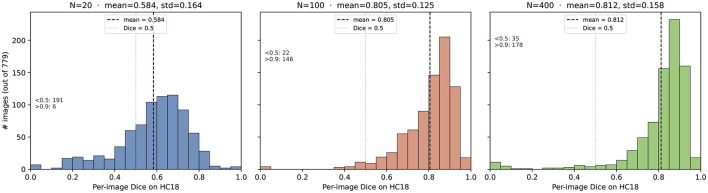
Per-image Dice distribution on HC18 (PSFHS-trained F-TPA-SAM2, seed 1, *N*_test_ = 779, no fine-tuning) for three label budgets. Dashed: mean Dice; dotted: Dice = 0.5. The bimodal shape—a non-trivial near-perfect subset (178/779 with Dice > 0.9 at *N* = 400) and a failure tail (35/779 with Dice < 0.5)—reflects inter-machine and gestational-age domain shift. HC18 masks are ellipse-derived, which may further lower measured Dice even when boundaries are qualitatively reasonable.

SAM 2 box with an Oracle bbox reaches 0.904 on HC18, but at the cost of requiring a bounding box, the clinician would have to draw. The corresponding per-image Dice distributions across N in {20, 100, 400} are shown in Figure 7.

### 3.5 Qualitative examples

**Figure 8:**
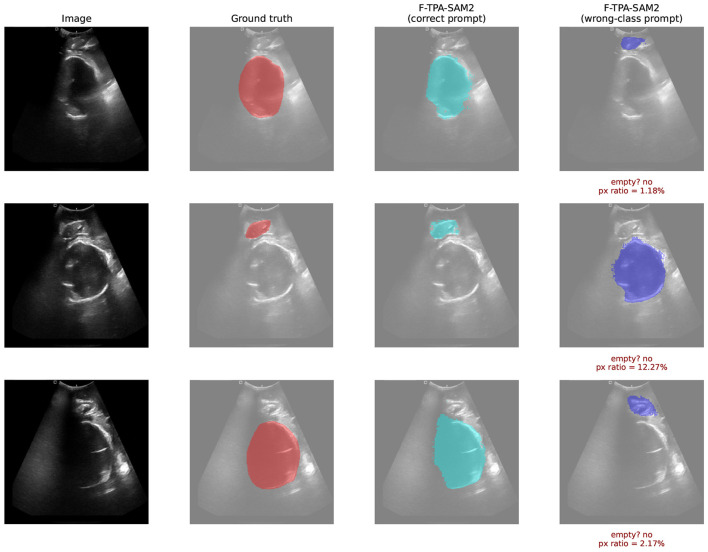
Qualitative PSFHS test examples. Column 3: F-TPA-SAM2 with the correct prompt produces tight masks. Column 4: under a wrong-class prompt, the model segments the *other* structure (consistent with Table 8). The fetal ultrasound images in Figure were adapted from the PSFHS dataset [Lu et al. (23); Zenodo record 10969427; CC BY 4.0].

Figure 8 shows three representative PSFHS test cases. Column 3 (F-TPA-SAM2 with the correct text prompt) produces masks that closely match the ground truth for both fetal head and pubic symphysis. Column 4 (wrong-class prompt) shows the model's wrong-structure response: the predicted mask has non-trivial area but overlaps with the *other* foreground structure, confirming the quantitative mask-area analysis.

**Figure 9:**
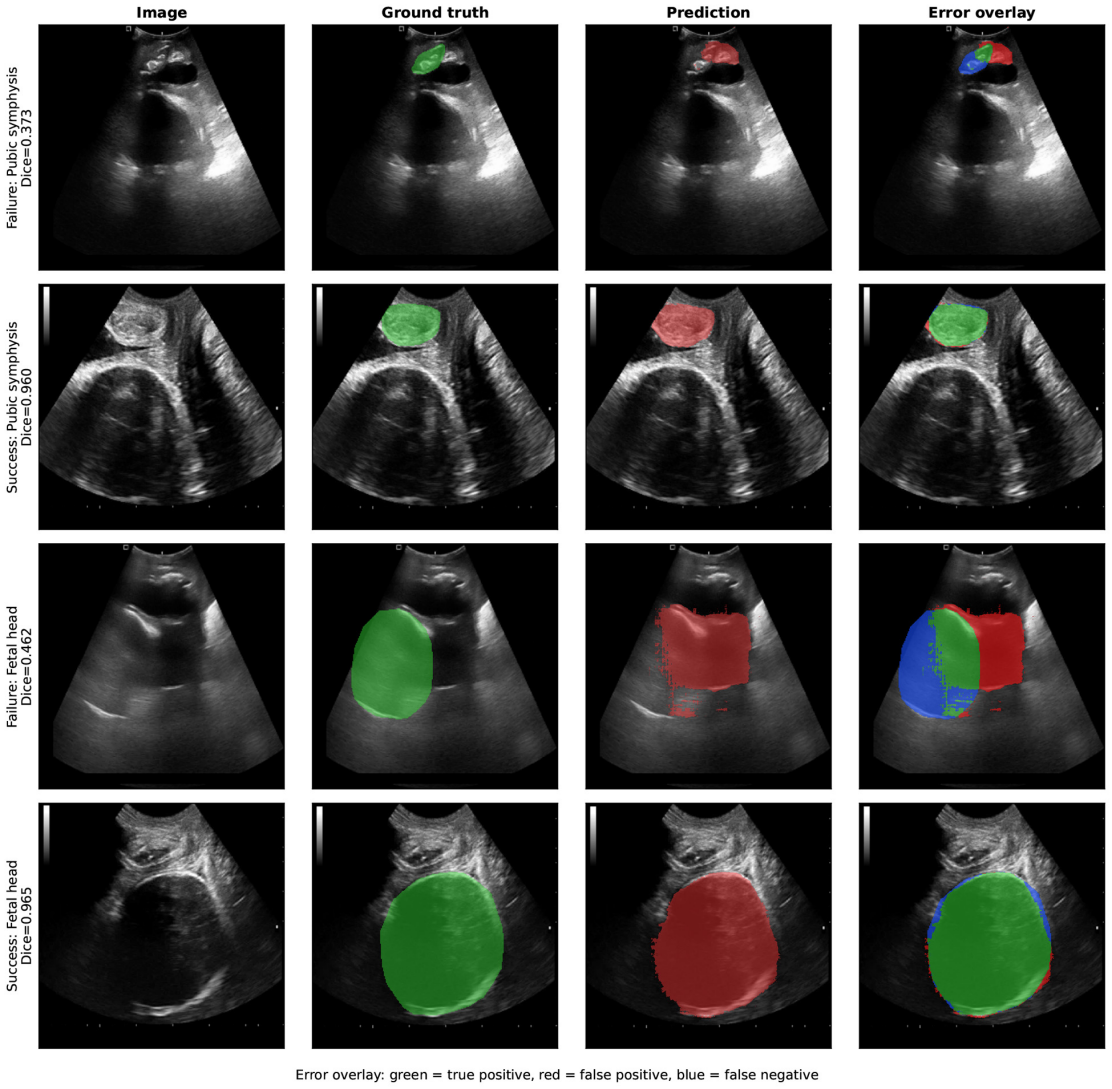
Fixed-rule PSFHS qualitative analysis. For each foreground class, the lowest- and highest-Dice test records from F-TPA-SAM2 (*N* = 400, seed 1) are shown. Error overlay: green = true positive, red = false positive, blue = false negative. The selection rule was fixed before visual inspection and is separate from the wrong-class prompt analysis in Figure 8. The fetal ultrasound images in Figure were adapted from the PSFHS dataset [Lu et al. (23); Zenodo record 10969427; CC BY 4.0].

Figure 9 supplements the prompt-swap examples with explicit PSFHS success and failure cases. The cases were selected by a fixed rule from the *N* = 400, seed-1 test predictions: the lowest- and highest-Dice record for each foreground class. The pubic symphysis failure predicts an adjacent echogenic region, while the fetal head failure is spatially displaced in a low-contrast field. These images illustrate, but do not prove, possible failure mechanisms. HC18 success and failure cases selected by the same performance-extreme principle are shown separately in Figure 6.

### 3.6 Computational cost and per-seed transparency

F-TPA-SAM2 has 0.871 M trainable parameters out of 645.4 M total (0.135%; see Table 2 in Section 2.2). Model inference was re-benchmarked with the text prompt pre-encoded, after 20 warm-up iterations, on 100 images repeated three times, with CUDA synchronization around every forward pass. At 1, 024 × 1, 024, bf16 and batch size 1 on an NVIDIA RTX PRO 6000 Blackwell Server Edition, mean latency was 0.0273 ± 0.0005 s per image (36.6 frames/s); the three repeat means were 0.02734, 0.02730, and 0.02730 s. Peak allocated and reserved GPU memory were 2.77 and 2.98 GiB, respectively. These measurements exclude data loading, preprocessing, display, model loading, and text encoding, and use high-end server hardware. They therefore characterize model-forward cost only, and do not establish real-time performance or deployment feasibility on a clinical ultrasound workstation. Training one (*N*, seed) configuration takes 20–50 min depending on *N*.

For reproducibility, we report all per-seed Dice values in Table 6 (test split, used for headline numbers) and Table 7 (validation split, used only for early stopping).

## 4 Discussion

### 4.1 Main findings

This study set out to test whether text prompts are actually being used to produce intrapartum fetal ultrasound segmentation, or whether the adapter silently learns a class-agnostic shape template. Four findings, in combination, answer this question for the two-class PSFHS setting.

**(i) Category-conditioned segmentation works at modest label budgets, but pretrained text is not necessary in this setting**. F-TPA-SAM2 reaches 0.811 Dice at *N* = 100 and 0.859 at *N* = 400 on the PSFHS test split. However, the parameter-matched no-text A0LR and fixed random class-code controls are not clearly different from F-TPA-SAM2 at either budget. The original gap to A0 reflected a confounded difference in capacity and parameterization, and cannot support a language-benefit claim. At *N* = 20, the original variants plateau at the no-information baseline; we do not claim utility there.

**(ii) The category-conditioned input is actually used, but this is not equivalent to pre-trained semantic understanding**. Within the two trained classes, under wrong-class prompts the predicted mask does not vanish (empty-mask rate = 0%) and matches the *opposite*-class ground truth at 0.859 ± 0.003 Dice—identical to the normal-prompt Dice on the requested target, with the same per-class breakdown (0.898 on fetal head, 0.820 on pubic symphysis), and exceeding the requested-class Dice on 100% of test images. This rules out one class-agnostic output for the two trained classes: the adapter follows the supplied category code to its trained another target. Blank or previously unseen random embeddings at inference disrupt this learned code. Conversely, distinct random vectors used consistently during both the training and testing studies as well as text embeddings. Therefore, the combined evidence supports stable category coding, not a claim that the pre-trained language semantics are causal or necessary. Open-vocabulary understanding would additionally require synonym, paraphrase, and unseen-anatomy controls.

**(iii) The trained adapter generalizes—partially—across datasets**. PSFHS-trained F-TPA-SAM2 produces 0.812 ± 0.157 Dice on HC18 fetal head with no fine-tuning. The mean is non-trivial, given the inter-machine and gestational-age domain shift, but the bimodal per-image distribution—178/779 images above 0.9 Dice and 35/779 below 0.5—is itself a finding. We do not read this as deployment-ready evidence.

**(iv) The matched supervised comparison depends on label budget**. F-TPA-SAM2 exceeds a from-scratch U-Net at *N* = 100, with a paired difference of 0.053 Dice (95% CI 0.006–0.085). At *N* = 400 the U-Net is numerically higher, but the paired CI includes zero. This is consistent with a benefit from the frozen pre-trained image backbone in the lower-label regime, while a standard supervised network becomes competitive as annotations increase. It does not establish the general superiority of F-TPA-SAM2 over task-specific supervised segmentation.

### 4.2 Workflow trade-off vs. oracle bounding boxes

Throughout our experiments, the Oracle-bbox SAM 2 baseline beats F-TPA-SAM2 by 0.018 Dice on PSFHS and 0.092 on HC18. This is the wrong reference frame for a clinical workflow claim. Drawing an accurate bounding box during routine sonography is itself a non-trivial expert intervention, and the practical alternative to a text prompt is manual spatial prompting by the sonographer. Obstetric ultrasound is inherently a *real-time dynamic* examination: the operator continuously sweeps the transducer to locate standard planes, making it impractical to pause, draw a bounding box on a frozen frame, and resume scanning. A text prompt—whether a system default or a voice-triggered command—can be supplied once before scanning begins and is applied to every incoming frame without interrupting the workflow. F-TPA-SAM2 is 0.018 Dice below this oracle-bbox reference on PSFHS while replacing the box with a fixed text string. The present study does not determine whether that numerical difference is acceptable in practice, or whether text input improves workflow efficiency. Those questions require a prospective user study measuring accuracy, time and cognitive burden.

### 4.3 Potential clinical implications and translational pathway

The clinical interest in text-prompted fetal segmentation is not about being faster on a leaderboard, but about the hypothetical ability to keep the operator's hands free during a time-sensitive examination. Three potential clinical scenarios motivate this design.

#### 4.3.1 Hypothetical intrapartum monitoring scenario

Repeated PS-FH distance and angle-of-progression measurements are routinely performed during the second stage of labor (2); if a text-prompted segmentation module were embedded in the ultrasound machine, and it could be triggered once at the start of monitoring, and then return per-frame segmentations of both structures throughout the examination, freeing the obstetrician to focus on probe positioning and clinical judgement. This scenario is presented as a motivation for future workflow studies, not as an established clinical claim.

#### 4.3.2 Hypothetical mid-trimester screening scenario

The ISUOG mid-trimester scanning guidelines (27) mandate measurement of several biometric parameters (head circumference, abdominal circumference, and femur length). A text-prompted interface could let the sonographer move between targets by words selected from the reporting template rather than by drawing new ones geometric prompts on each plane. We do not evaluate this scenario; it is included only as a possible future application.

#### 4.3.3 Hypothetical multi-site research scenario

A category-conditioned adapter exposes target selection as an input within the set of anatomies used for training. New anatomical targets would still require labeled adaptation data and re-training. Any multi-site infrastructure benefit is therefore hypothetical and is not a demonstrated result.

The translational pathway we envisage has four steps: (1) the present methodological evidence that text actually controls the segmentation output within trained classes; (2) prospective validation on a multi-center fetal ultrasound cohort with patient-stratified splits and annotation by multiple sonographers; (3) a sonographer-in-the-loop reader study comparing text input, geometric input, and no input on clinical time and cognitive load; and (4) regulatory-grade validation under the local medical-device framework. The current paper addresses step (1); steps (2)–(4) are explicitly the responsibility of subsequent study.

We emphasize that no clinician evaluation, workflow study, time-saving analysis, or user-interaction experiment, is reported here. Any statement about clinical utility should therefore be read as a hypothesis for future investigation rather than as an established finding.

### 4.4 Limitations

We summarize the limitations that bound the scope of our claims.

#### 4.4.1 Class-conditional, not open-vocabulary, and semantics

The negative controls show that the adapter follows the text prompt to select between the two trained anatomical classes, but they do not test open-vocabulary semantic understanding. The wrong-class control is a class-swap between two trained tokens, not an evaluation on synonyms, paraphrased prompts, spelling perturbations, negations, or unseen anatomical terms.

#### 4.4.2 Architectural-control fairness

The original A0 comparison is confounded by its 2.10 M versus 0.871 M trainable parameters and different dense-prompt parameterization. We therefore added A0LR and fixed random class codes; both are statistically compatible with F-TPA-SAM2 at *N* = 100 and *N* = 400. These controls remove the basis for attributing the original A0 gap to the text encoder. A deterministic two-class label swap was not used as a semantic evidence because it is a perfectly learnable reverse code; a meaningful sample-level random-label experiment would test robustness to corrupted supervision rather than the necessity of language semantics.

#### 4.4.3 Sparse-only generalization

Our A1 control uses two learnable sparse tokens; it tells us that this specific sparse-only design is insufficient under the frozen SAM 2 decoder, not that all sparse-prompt designs fail. Richer sparse-token variants and mixed sparse-dense designs are left to future study.

#### 4.4.4 Partial external comparison

Our only direct comparison against the text-guided medical SAM family is with MedCLIP-SAMv2 (16), and only under an Oracle-bounding-box reference. A saliency-driven reproduction at matched label budgets and a faithful Grounding DINO-US-SAM (19) re-training remain outside the present comparison. We added a standard supervised U-Net under the same split, label budgets and seeds; it is lower at *N* = 100 and not clearly different at *N* = 400. This single matched baseline does not substitute for a broad benchmark of task-specific fetal-US architectures. Published results from such methods remain non-direct context because their splits, targets, and annotation budgets differ.

#### 4.4.5 Two fetal anatomies; image-level split

Our experiments cover pubic symphysis and fetal head; the cross-dataset transfer to HC18 addresses only the fetal head. We therefore scope our claim to two-structure intrapartum fetal-US segmentation. The wrong-class control is a swap between these two trained class tokens, not an open-vocabulary test of synonyms, paraphrased prompts, or unseen anatomical terms. The public PSFHS release we use ships frame-level identifiers only and does not expose patient or video identifiers; our 70/15/15 split is therefore image-level, and we cannot rule out patient-level or sequence-level leakage between train, validation, and test splits. Because frames originating from the same acquisition may appear in both training and testing subsets, the reported Dice values may be optimistic relative to a fully patient-independent evaluation. This is not a statistical upper bound: neither the direction nor the magnitude of the potential bias can be quantified without the identifiers that are absent from the public release. A patient-stratified split is therefore required for prospective clinical validation.

#### 4.4.6 Annotation-style differences

HC18 masks are derived from ellipse fits to fetal-head circumference annotations, whereas PSFHS masks are pixel-level annotations of two anatomies. The cross-dataset Dice we report on HC18 may therefore be lower than the qualitative segmentation quality suggests.

#### 4.4.7 Static-image evaluation only

We do not exercise SAM 2's video memory. B-mode ultrasound is a video modality, and text-prompted cine-loop propagation is a natural extension that we leave to future study.

#### 4.4.8 Encoder ablation depth

We compare three encoders at *N* = 100 with three seeds and report paired bootstrap CIs. The statistically distinguishable FetalCLIP– BiomedCLIP difference is only 0.009 Dice, FetalCLIP is not clearly different from OpenCLIP, and fixed random codes are competitive. A wider encoder panel or a larger anatomy vocabulary could yield different conclusions.

### 4.5 Conclusion

Through matched controls, we show that the 0.135%-trainable F-TPA-SAM2 uses a category-conditioned input to select between two trained PSFHS targets: a wrong-class prompt redirects the prediction to the other class, whereas the model does not produce one class-agnostic mask. However, A0LR and fixed random class codes perform comparably, so this study does not demonstrate that pre-trained language semantics are necessary or beneficial for two-class segmentation. F-TPA-SAM2 outperforms a from-scratch U-Net at *N* = 100 but is not clearly different at *N* = 400, and same-anatomy HC18 transfer retains substantial mean performance with a visible failure tail. We do not claim open-vocabulary understanding, superiority to the broader text-guided medical SAM family, or generalization beyond the two trained anatomies. Broader anatomy, patient-stratified prospective validation, matched external reproduction and cine-loop evaluation remain a future study.

## Data Availability

The datasets analyzed in this study are publicly available: PSFHS at Zenodo, record 10969427 (https://doi.org/10.5281/zenodo.10969427), and HC18 at Zenodo, record 1322001 (https://doi.org/10.5281/zenodo.1322001). The training code and trained model weights that support the findings of this study are available from the corresponding author upon reasonable request.

## References

[1] Salomon LJ Alfirevic Z Da Silva Costa F Deter RL Figueras F Ghi T . ISUOG Practice Guidelines (updated): performance of the routine mid-trimester fetal ultrasound scan. Ultras Obstetr Gynecol. (2019) 53:715–23. doi: 10.1002/uog.20272 35592929

[2] Ghi T EggebrøT Lees C Kalache K Rozenberg P Youssef A . ISUOG practice guidelines: intrapartum ultrasound. Ultras Obstetr Gynecol. (2018) 52:128–39. doi: 10.1002/uog.19072 29974596

[3] Tutschek B Torkildsen EA Eggebø TM. Comparison between ultrasound parameters and clinical examination to assess fetal head station in labor. Ultrasound Obstet Gynecol. (2013) 41:425–9. doi: 10.1002/uog.12422 23371409

[4] Wang F Silvestre G Curran KM. MiTU-Net: A fine-tuned U-Net with SegFormer backbone for segmenting pubic symphysis-fetal head. arXiv preprint arXiv:2401.15513. (2024).

[5] Wang F Silvestre G Curran KM. Dual agreement consistency learning with foundation models for semi-supervised fetal heart ultrasound segmentation and diagnosis. In: 2026 IEEE international symposium on biomedical imaging (ISBI) (2026). doi: 10.1109/ISBI61048.2026.11515399

[6] Zhuang T Hu S Luo Y Zhang Z Li Y . Synergistic foundation models for semi-supervised fetal cardiac ultrasound analysis: SAM-Med2D boundary refinement and DINOv3 semantic enhancement. arXiv preprint arXiv:2605.19799. (2026).

[7] Kirillov A Mintun E Ravi N Mao H Rolland C Gustafson L . Segment anything. In: Proceedings of the IEEE/CVF international conference on computer vision (ICCV) (2023). p. 4015–4026. doi: 10.1109/ICCV51070.2023.00371

[8] Ravi N Gabeur V Hu YT Hu R Ryali C Ma T . SAM 2: segment anything in images and videos. In: Proceedings of the international conference on learning representations (ICLR) (2025).

[9] Mazurowski MA Dong H Gu H Yang J Konz N . Segment anything model for medical image analysis: an experimental study. Med Image Anal. (2023) 89:102918. doi: 10.1016/j.media.2023.102918 37595404 PMC10528428

[10] Ma J He Y Li F Han L You C Wang B. Segment anything in medical images. Nat Commun. (2024) 15:654. doi: 10.1038/s41467-024-44824-z 38253604 PMC10803759

[11] Cheng J Ye J Deng Z Chen J Li T Wang H . SAM-Med2D. arXiv preprint arXiv:2308.16184. (2023).

[12] Wu J Ji W Liu Y Fu H Xu M Xu Y . Medical SAM adapter: adapting segment anything model for medical image segmentation. Med Image Anal. (2025) 102:103547. doi: 10.1016/j.media.2025.103547 40121809

[13] Liu Z Dong S Li B Yang Y Ruan J . BALR-SAM: boundary-aware low-rank adaptation of SAM for resource-efficient medical image segmentation. arXiv preprint arXiv:2509.24204. (2025).

[14] Xie Z Guan B Jiang W Yi M Ding Y . PA-SAM: prompt adapter SAM for high-quality image segmentation. In: 2024 IEEE international conference on multimedia and expo (ICME) (2024). doi: 10.1109/ICME57554.2024.10687602

[15] Koleilat T Asgariandehkordi H Rivaz H Xiao Y. MedCLIP-SAM: bridging text and image towards universal medical image segmentation. In: International conference on medical image computing and computer-assisted intervention (MICCAI). Springer (2024). p. 643–653. doi: 10.1007/978-3-031-72390-2_60

[16] Koleilat T Asgariandehkordi H Rivaz H Xiao Y. MedCLIP-SAMv2: towards universal text-driven medical image segmentation. Med Image Anal. (2025) 106:103749. doi: 10.1016/j.media.2025.103749 40779830

[17] Zhang W Luo L He M Hai J Ye J. DescriptorMedSAM: language-image fusion with multi-aspect text guidance for medical image segmentation. Sci Rep. (2026) 16:3758. doi: 10.1038/s41598-025-33843-5 41526554 PMC12852884

[18] Huang S Liang H Wang Q Zhong C Zhou Z Shi M. SEG-SAM: semantic-guided SAM for unified medical image segmentation. arXiv preprint arXiv:2412.12660. (2024).

[19] Rasaee H Koleilat T Rivaz H. Grounding DINO-US-SAM: text-prompted multiorgan segmentation in ultrasound with LoRA-tuned vision-language models. IEEE Trans Ultrason Ferroelectr Freq Control. (2025) 72:1414–25. doi: 10.1109/TUFFC.2025.3605285 40892652

[20] Spiegler P Koleilat T Harirpoush A Miller CS Rivaz H Kersten-Oertel M . TextSAM-EUS: text prompt learning for SAM to accurately segment pancreatic tumor in endoscopic ultrasound. In: Proceedings of the IEEE/CVF international conference on computer vision (ICCV) workshops (2025). p. 959–968. doi: 10.1109/ICCVW69036.2025.00104

[21] Maani F Saeed N Saleem T Farooq Z Alasmawi H . FetalCLIP: a visual-language foundation model for fetal ultrasound image analysis. arXiv preprint arXiv:2502.14807. (2025). 10.1038/s41746-026-02907-9 PMC13642926 42321373

[22] van den Heuvel TLA de Bruijn D de Korte CL Ginneken BV. Automated measurement of fetal head circumference using 2D ultrasound images. PLoS ONE. (2018) 13:e0200412. doi: 10.1371/journal.pone.0200412 30138319 PMC6107118

[23] Lu Y Zhou M Zhi D Zhou M Jiang X Qiu R . The JNU-IFM dataset for segmenting pubic symphysis-fetal head. Data in Brief . (2022) 41:107904. doi: 10.1016/j.dib.2022.107904 35198683 PMC8842023

[24] Ronneberger O Fischer P Brox T. U-Net: convolutional networks for biomedical image segmentation. In: Medical image computing and computer-assisted intervention-MICCAI 2015. Springer (2015). p. 234–241. doi: 10.1007/978-3-319-24574-4_28

[25] Zhang S Xu Y Usuyama N Xu H Bagga J Tinn R . A multimodal biomedical foundation model trained from fifteen million image-text pairs. NEJM AI. (2024) 2:AIoa2400640. doi: 10.1056/AIoa2400640

[26] Cherti M Beaumont R Wightman R Wortsman M Ilharco G Gordon C . Reproducible scaling laws for contrastive language-image learning. In: Proceedings of the IEEE/CVF conference on computer vision and pattern recognition (CVPR) (2023). p. 2818–2829. doi: 10.1109/CVPR52729.2023.00276

[27] Salomon LJ Alfirevic Z Berghella V Bilardo CM Chalouhi GE Da Silva Costa F . ISUOG Practice Guidelines (updated): performance of the routine mid-trimester fetal ultrasound scan. Ultrasound Obstetr Gynecol. (2022) 59:840–56. doi: 10.1002/uog.24888 35592929

